# Supplementary material for: Proteomic analysis of Masson pine with high resistance to pine wood nematodes
Source: PLoS One. 2022 Aug 12;17(8):e0273010. doi: 10.1371/journal.pone.0273010 (PMC9374249; doi:10.1371/journal.pone.0273010)
Supplement: S1 Table — (DOCX) [file pone.0273010.s001.docx]

**Supporting Data Table 1. The protein numbers of GO functional analysis with up-regulation in resistant seedlings**

| **GO Terms Level 1** | **GO Terms Level 2** | **Number of proteins** |
| --- | --- | --- |
| Biological Process | cellular process | 14 |
| Biological Process | metabolic process | 11 |
| Biological Process | response to stimulus | 8 |
| Biological Process | cellular component organization or biogenesis | 8 |
| Biological Process | biological regulation | 6 |
| Biological Process | multicellular organismal process | 3 |
| Biological Process | developmental process | 3 |
| Biological Process | signaling | 2 |
| Biological Process | localization | 2 |
| Biological Process | reproduction | 1 |
| Biological Process | reproductive process | 1 |
| Biological Process | rhythmic process | 1 |
| Biological Process | multi-organism process | 1 |
| Cellular Component | cell | 22 |
| Cellular Component | organelle | 15 |
| Cellular Component | membrane | 9 |
| Cellular Component | cell junction | 6 |
| Cellular Component | membrane-enclosed lumen | 5 |
| Cellular Component | protein-containing complex | 5 |
| Cellular Component | symplast | 5 |
| Molecular Function | catalytic activity | 11 |
| Molecular Function | binding | 8 |
