# Supplementary material for: Proteomic analysis of Masson pine with high resistance to pine wood nematodes
Source: PLoS One. 2022 Aug 12;17(8):e0273010. doi: 10.1371/journal.pone.0273010 (PMC9374249; doi:10.1371/journal.pone.0273010)
Supplement: S2 Table — (DOCX) [file pone.0273010.s002.docx]

**Supporting Data Table 2. The protein numbers of GO functional analysis with down-regulation in resistant seedlings**

| **GO Terms Level 1** | **GO Terms Level 2** | **Number of proteins** |
| --- | --- | --- |
| Biological Process | cellular process | 32 |
| Biological Process | metabolic process | 31 |
| Biological Process | response to stimulus | 28 |
| Biological Process | biological regulation | 23 |
| Biological Process | multi-organism process | 14 |
| Biological Process | developmental process | 11 |
| Biological Process | multicellular organismal process | 10 |
| Biological Process | cellular component organization or biogenesis | 7 |
| Biological Process | localization | 6 |
| Biological Process | reproduction | 5 |
| Biological Process | reproductive process | 5 |
| Biological Process | other | 7 |
| Cellular Component | cell | 50 |
| Cellular Component | organelle | 30 |
| Cellular Component | membrane | 18 |
| Cellular Component | extracellular region | 6 |
| Cellular Component | protein-containing complex | 6 |
| Cellular Component | cell junction | 4 |
| Cellular Component | membrane-enclosed lumen | 4 |
| Cellular Component | symplast | 4 |
| Cellular Component | other | 1 |
| Molecular Function | catalytic activity | 36 |
| Molecular Function | binding | 12 |
| Molecular Function | molecular function regulator | 6 |
| Molecular Function | catalytic activity, acting on a protein | 6 |
| Molecular Function | transporter activity | 3 |
| Molecular Function | antioxidant activity | 3 |
| Molecular Function | structural molecule activity | 2 |
| Molecular Function | molecular transducer activity | 1 |
| Molecular Function | molecular carrier activity | 1 |
