# Supplementary material for: Proteomic analysis of Masson pine with high resistance to pine wood nematodes
Source: PLoS One. 2022 Aug 12;17(8):e0273010. doi: 10.1371/journal.pone.0273010 (PMC9374249; doi:10.1371/journal.pone.0273010)
Supplement: S3 Table — (DOCX) [file pone.0273010.s003.docx]

**Supporting Date Table 3. List of the up-regulated and down-regulated proteins in resistant vs. susceptible strains**

| **Protein accession** | **Protein description** | **R/S Ratio** | **R/S P value** | **Regulated Type** |
| --- | --- | --- | --- | --- |
| comp3199_c0_seq1_m.5970_g.5970 | Peroxisomal biogenesis factor, putative | 5.748 | 2.695E-04 | Up |
| comp15407_c0_seq1_m.20543_g.20543 | phospholipase A1-Igamma3, chloroplastic-like | 5.685 | 8.733E-06 | Up |
| comp10059_c0_seq1_m.1041_g.1041 | protein LOW PSII ACCUMULATION 1, chloroplastic isoform X1 | 5.038 | 3.345E-05 | Up |
| comp2151_c0_seq3_m.6243_g.6243 | TSA: Wollemia nobilis Ref_Wollemi_Transcript_3779_2119 transcribed RNA sequence | 2.144 | 3.508E-05 | Up |
| comp8228_c0_seq1_m.7956_g.7956 | probable aspartyl protease At4g16563 | 1.9 | 3.540E-04 | Up |
| comp9708_c0_seq5_m.7975_g.7975 | Uncharacterized protein | 1.845 | 2.042E-04 | Up |
| comp34443_c0_seq2_m.4923_g.4923 | TSA: Wollemia nobilis Ref_Wollemi_Transcript_21034_833 transcribed RNA sequence | 1.774 | 9.439E-04 | Up |
| comp16524_c0_seq2_m.2127_g.2127 | PHP (Fragment) | 1.753 | 1.205E-02 | Up |
| comp7090_c0_seq1_m.3857_g.3857 | TSA: Wollemia nobilis Ref_Wollemi_Transcript_18565_2317 transcribed RNA sequence | 1.681 | 3.035E-04 | Up |
| comp53_c0_seq1_m.11270_g.11270 | Histone H3 | 1.672 | 1.489E-03 | Up |
| comp50441_c0_seq1_m.9660_g.9660 | Adenosylhomocysteinase | 1.613 | 6.096E-05 | Up |
| comp13287_c0_seq1_m.13169_g.13169 | myosin-11-like | 1.609 | 1.469E-05 | Up |
| comp8918c0_seq2_m.8473_g.473 | Putative TIR-NBS-LRR protein | 1.599 | 6.987E-06 | Up |
| comp5207_c0_seq2_m.18603_g.18603 | TMV resistance protein N-like isoform X2 | 1.58 | 8.582E-06 | Up |
| comp1952_c0_seq8_m.11774_g.11774 | PDC2 | 1.564 | 1.116E-03 | Up |
| comp3852_c0_seq1_m.5479_g.5479 | Uncharacterized protein | 1.524 | 2.642E-04 | Up |
| comp23587_c0_seq2_m.16549_g.16549 | Sucrose synthase | 1.519 | 1.529E-05 | Up |
| comp501_c0_seq2_m.10779_g.10779 | heterogeneous nuclear ribonucleoprotein Q | 1.507 | 1.218E-05 | Up |
| comp22020_c0_seq1_m.16371_g.16371 | Beta-galactosidase | 1.5 | 8.829E-03 | Up |
| comp6525_c0_seq1_m.3765_g.3765 | RING-type E3 ubiquitin transferase | 1.465 | 4.185E-04 | Up |
| comp2679_c0_seq1_m.5399_g.5399 | TSA: Wollemia nobilis Ref_Wollemi_Transcript_7847_1318 transcribed RNA sequence | 1.46 | 4.352E-04 | Up |
| comp7682_c0_seq1_m.3500_g.3500 | Cytochrome P450 CYP867A (Fragment) | 1.449 | 3.227E-02 | Up |
| comp1939_c0_seq1_m.11032_g.11032 | serine/arginine-rich splicing factor RSZ21A isoform X2 | 1.435 | 4.024E-03 | Up |
| comp6615_c0_seq6_m.4419_g.4419 | probable nucleolar protein 5-2 | 1.404 | 1.379E-04 | Up |
| comp7028_c0_seq1_m.3978_g.3978 | RNA helicase | 1.399 | 5.144E-03 | Up |
| comp11410_c0_seq1_m.457_g.457 | Aspartic proteinase 4 (Fragment) | 1.39 | 1.509E-02 | Up |
| comp23924_c0_seq1_m.16450_g.16450 | Uncharacterized protein | 1.383 | 2.466E-02 | Up |
| comp68_c0_seq1_m.11926_g.11926 | TSA: Wollemia nobilis Ref_Wollemi_Transcript_8439_1825 transcribed RNA sequence | 1.374 | 1.396E-04 | Up |
| comp9874_c0_seq1_m.8682_g.8682 | universal stress protein PHOS32-like | 1.362 | 7.198E-04 | Up |
| comp68_c0_seq2_m.11927_g.11927 | TSA: Wollemia nobilis Ref_Wollemi_Transcript_8439_1825 transcribed RNA sequence | 1.348 | 9.411E-04 | Up |
| comp11257_c0_seq1_m.902_g.902 | Phytochromobilin:ferredoxin oxidoreductase chloroplastic | 1.345 | 5.177E-06 | Up |
| comp32296_c0_seq1_m.14372_g.14372 | Ubiquitin-like modifier-activating enzyme 5 (Fragment) | 1.342 | 4.637E-04 | Up |
| comp8845_c0_seq1_m.9492_g.9492 | plant intracellular Ras-group-related LRR protein 1-like | 1.338 | 1.002E-03 | Up |
| comp17545_c0_seq1_m.1602_g.1602 | Uncharacterized protein | 1.337 | 9.285E-04 | Up |
| comp6899_c0_seq2_m.4211_g.4211 | TSA: Wollemia nobilis Ref_Wollemi_Transcript_14766_2641 transcribed RNA sequence | 1.333 | 1.880E-04 | Up |
| comp28188_c0_seq1_m.19779_g.19779 | Protein GAL3 | 1.329 | 5.411E-03 | Up |
| comp5416_c0_seq1_m.18779_g.18779 | Histone H2A | 1.319 | 2.265E-02 | Up |
| comp2414_c0_seq1_m.6401_g.6401 | Myb family transcription factor PHL3 | 1.313 | 2.613E-04 | Up |
| comp9641_c0_seq1_m.8266_g.8266 | RNA-binding protein | 1.31 | 2.377E-02 | Up |
| comp6899_c0_seq1_m.4210_g.4210 | TSA: Wollemia nobilis Ref_Wollemi_Transcript_14766_2641 transcribed RNA sequence | 1.308 | 1.911E-04 | Up |
| comp1216_c0_seq9_m.11065_g.11065 | Protein VEIN PATTERNING 1 | 1.305 | 2.120E-03 | Up |
| comp6868_c0_seq1_m.3569_g.3569 | TSA: Wollemia nobilis Ref_Wollemi_Transcript_22579_2628 transcribed RNA sequence | 1.303 | 1.547E-03 | Up |
| comp2628_c0_seq2_m.6851_g.6851 | Oxidative stress 3, putative isoform 1 | 0.769 | 5.302E-04 | Down |
| comp2923_c0_seq1_m.6459_g.6459 | TSA: Wollemia nobilis Ref_Wollemi_Transcript_26238_1798 transcribed RNA sequence | 0.768 | 5.252E-03 | Down |
| comp19763_c0_seq1_m.14882_g.14882 | 3-hydroxyisobutyrate dehydrogenase | 0.767 | 7.584E-04 | Down |
| comp8256_c0_seq2_m.8552_g.8552 | Peroxidase | 0.767 | 4.557E-04 | Down |
| comp14727_c0_seq1_m.20531_g.20531 | Microtubule-associated protein RP/EB family member 1C-like protein | 0.765 | 5.858E-03 | Down |
| comp654_c0_seq1_m.10958_g.10958 | Acyl carrier protein | 0.765 | 7.359E-04 | Down |
| comp430_c0_seq2_m.10550_g.10550 | TSA: Wollemia nobilis Ref_Wollemi_Transcript_14739_2197 transcribed RNA sequence | 0.761 | 3.039E-04 | Down |
| comp830_c0_seq1_m.10881_g.10881 | BnaA03g18340D protein | 0.761 | 1.202E-04 | Down |
| comp1898_c0_seq1_m.11084_g.11084 | BnaA08g05440D protein | 0.756 | 6.803E-03 | Down |
| comp2198_c0_seq4_m.5641_g.5641 | Putative flavoprotein-containing polyamine oxidase (Fragment) | 0.756 | 8.372E-05 | Down |
| comp19952_c0_seq1_m.14723_g.14723 | Purple acid phosphatase | 0.755 | 4.014E-04 | Down |
| comp12992_c0_seq1_m.13638_g.13638 | Ornithine cyclodeaminase/mu-crystallin | 0.754 | 8.638E-04 | Down |
| comp4875_c0_seq1_m.18666_g.18666 | Cytochrome b561 and DOMON domain-containing protein | 0.754 | 2.958E-04 | Down |
| comp11800_c0_seq1_m.59_g.59 | Peroxisomal nicotinamide adenine dinucleotide carrier-like protein isoform X1 | 0.753 | 3.849E-03 | Down |
| comp11887_c0_seq1_m.1354_g.1354 | ATVPS33 vacuolar protein sorting 33 | 0.753 | 2.511E-02 | Down |
| comp7037_c0_seq1_m.2690_g.2690 | Protein kinase domain | 0.753 | 1.705E-02 | Down |
| comp11802_c0_seq1_m.961_g.961 | TSA: Wollemia nobilis Ref_Wollemi_Transcript_15793_2117 transcribed RNA sequence | 0.752 | 1.025E-02 | Down |
| comp19857_c0_seq1_m.15072_g.15072 | Uncharacterized protein | 0.752 | 9.462E-04 | Down |
| comp205_c1_seq1_m.11463_g.11463 | Nucleoside diphosphate kinase | 0.751 | 2.944E-03 | Down |
| comp1024_c0_seq1_m.11249_g.11249 | Putative NADH:ubiquinone reductase (H(+)-translocating) | 0.748 | 2.794E-04 | Down |
| comp10582_c0_seq1_m.698_g.698 | Beta-hexosaminidase | 0.747 | 1.639E-04 | Down |
| comp10202_c0_seq1_m.99_g.99 | Purple acid phosphatase | 0.746 | 2.593E-02 | Down |
| comp589_c0_seq1_m.10339_g.10339 | Putative proline-rich arabinogalactan protein (Fragment) | 0.744 | 7.896E-04 | Down |
| comp13501_c0_seq1_m.12645_g.12645 | E3 ubiquitin-protein ligase HOS1 isoform X1 | 0.742 | 2.419E-03 | Down |
| comp4367_c0_seq5_m.17814_g.17814 | Pectinesterase | 0.742 | 1.780E-05 | Down |
| comp4755_c0_seq2_m.18674_g.18674 | TSA: Wollemia nobilis Ref_Wollemi_Transcript_25234_1881 transcribed RNA sequence | 0.741 | 1.363E-03 | Down |
| comp9038_c0_seq2_m.8437_g.8437 | TSA: Wollemia nobilis Ref_Wollemi_Transcript_13270_2450 transcribed RNA sequence | 0.74 | 3.911E-03 | Down |
| comp19916_c0_seq1_m.14629_g.14629 | Circadian locomoter output cycles protein like | 0.737 | 3.074E-03 | Down |
| comp1523_c0_seq2_m.10454_g.10454 | Pectinesterase | 0.732 | 5.670E-05 | Down |
| comp31814_c0_seq1_m.10074_g.10074 | TSA: Wollemia nobilis Ref_Wollemi_Transcript_778_1460 transcribed RNA sequence | 0.728 | 7.043E-04 | Down |
| comp10056_c0_seq1_m.548_g.548 | TSA: Wollemia nobilis Ref_Wollemi_Transcript_1607_1920 transcribed RNA sequence | 0.726 | 3.341E-04 | Down |
| comp140_c0_seq4_m.10949_g.10949 | TSA: Wollemia nobilis Ref_Wollemi_Transcript_18766_1911 transcribed RNA sequence | 0.726 | 2.653E-03 | Down |
| comp17582_c0_seq1_m.1703_g.1703 | alpha-L-fucosidase 2 | 0.723 | 1.654E-04 | Down |
| comp1143_c0_seq2_m.10311_g.10311 | Putative sinapyl alcohol dehydrogenase (Fragment) | 0.72 | 1.703E-03 | Down |
| comp6167_c0_seq1_m.2653_g.2653 | EF-hand domain | 0.713 | 1.556E-02 | Down |
| comp7152_c0_seq1_m.3222_g.3222 | Bis(5'-nucleosyl)-tetraphosphatase, symmetrical like | 0.709 | 1.476E-05 | Down |
| comp9663_c0_seq1_m.9189_g.9189 | TSA: Wollemia nobilis Ref_Wollemi_Transcript_18719_3499 transcribed RNA sequence | 0.709 | 3.736E-03 | Down |
| comp1837_c0_seq1_m.11599_g.11599 | Putative Heat shock protein DnaJ, cysteine-rich | 0.707 | 1.023E-03 | Down |
| comp1850_c0_seq1_m.11177_g.11177 | TSA: Wollemia nobilis Ref_Wollemi_Transcript_14637_2708 transcribed RNA sequence | 0.707 | 6.115E-04 | Down |
| comp5126_c0_seq1_m.18540_g.18540 | Protein DMR6-LIKE OXYGENASE 2-like protein | 0.707 | 4.739E-05 | Down |
| comp5943_c0_seq1_m.18725_g.18725 | Carboxypeptidase | 0.706 | 5.606E-04 | Down |
| comp18315_c0_seq1_m.14995_g.14995 | Triacylglycerol lipase SDP1 | 0.705 | 1.041E-04 | Down |
| comp26104_c0_seq1_m.15703_g.15703 | Late embryogenesis abundant protein LEA7-2 | 0.703 | 1.481E-04 | Down |
| comp2509_c0_seq1_m.5419_g.5419 | Class IV chitinase Chia4-Pa1 | 0.698 | 1.575E-05 | Down |
| comp10624_c0_seq1_m.999_g.999 | TSA: Wollemia nobilis Ref_Wollemi_Transcript_5736_1229 transcribed RNA sequence | 0.696 | 9.402E-03 | Down |
| comp8251_c0_seq1_m.7805_g.7805 | Alpha-mannosidase | 0.694 | 3.171E-04 | Down |
| comp20367_c0_seq1_m.7244_g.7244 | DNA repair helicase XPD isoform X2 | 0.691 | 6.114E-05 | Down |
| comp5943_c0_seq8_m.18730_g.18730 | Carboxypeptidase | 0.69 | 7.873E-04 | Down |
| comp4952_c0_seq2_m.17999_g.17999 | Putative ovule protein | 0.689 | 1.507E-03 | Down |
| comp347_c0_seq1_m.11701_g.11701 | Histone H2A | 0.686 | 2.513E-03 | Down |
| comp17987_c0_seq2_m.1860_g.1860 | Laccase | 0.683 | 6.604E-06 | Down |
| comp18815_c0_seq1_m.14756_g.14756 | NADH dehydrogenase [ubiquinone] 1 alpha subcomplex subunit 13-B | 0.682 | 3.538E-03 | Down |
| comp28045_c0_seq1_m.19710_g.19710 | 40S ribosomal protein S9 (Fragment) | 0.682 | 3.171E-03 | Down |
| comp12466_c0_seq1_m.12852_g.12852 | Alpha-mannosidase (Fragment) | 0.681 | 6.674E-04 | Down |
| comp7985_c0_seq2_m.3385_g.3385 | ABCB1 protein | 0.68 | 1.139E-04 | Down |
| comp3112_c0_seq2_m.6100_g.6100 | TSA: Wollemia nobilis Ref_Wollemi_Transcript_14249_2194 transcribed RNA sequence | 0.679 | 4.884E-03 | Down |
| comp3262_c0_seq1_m.5637_g.5637 | TSA: Wollemia nobilis Ref_Wollemi_Transcript_4268_1890 transcribed RNA sequence | 0.677 | 3.022E-03 | Down |
| comp36095_c0_seq1_m.4666_g.4666 | Uncharacterized protein | 0.677 | 6.908E-05 | Down |
| comp5732_c0_seq1_m.19202_g.19202 | TSA: Wollemia nobilis Ref_Wollemi_Transcript_22280_1481 transcribed RNA sequence | 0.676 | 8.163E-04 | Down |
| comp8443_c0_seq1_m.8852_g.8852 | Uncharacterized protein | 0.671 | 2.940E-02 | Down |
| comp11923_c0_seq1_m.695_g.695 | Xyloglucan endotransglucosylase/hydrolase | 0.67 | 1.832E-05 | Down |
| comp18721_c0_seq1_m.14842_g.14842 | Peroxidase | 0.666 | 2.134E-06 | Down |
| comp15642_c0_seq1_m.20683_g.20683 | Peroxidase | 0.665 | 1.777E-04 | Down |
| comp3825_c0_seq1_m.5513_g.5513 | TSA: Wollemia nobilis Ref_Wollemi_Transcript_8589_2154 transcribed RNA sequence | 0.665 | 3.762E-04 | Down |
| comp7221_c0_seq1_m.4337_g.4337 | Protein TIC | 0.65 | 1.355E-03 | Down |
| comp2649_c0_seq6_m.5545_g.5545 | TPR1 | 0.645 | 1.301E-04 | Down |
| comp17876_c0_seq1_m.1985_g.1985 | Glycosyltransferase | 0.639 | 4.532E-06 | Down |
| comp702_c0_seq1_m.11024_g.11024 | 60S acidic ribosomal protein family | 0.638 | 1.678E-02 | Down |
| comp4071_c0_seq1_m.18442_g.18442 | Beta-galactosidase | 0.63 | 3.157E-05 | Down |
| comp415_c0_seq2_m.11806_g.11806 | Histone H2A | 0.625 | 3.436E-05 | Down |
| comp5659_c0_seq10_m.17972_g.17972 | L-ascorbate oxidase homolog | 0.624 | 7.387E-06 | Down |
| comp1741_c0_seq1_m.11454_g.11454 | TSA: Wollemia nobilis Ref_Wollemi_Transcript_5294_1444 transcribed RNA sequence | 0.617 | 4.029E-05 | Down |
| comp5659_c0_seq5_m.17967_g.17967 | L-ascorbate oxidase-like protein | 0.6 | 1.238E-05 | Down |
| comp12951_c0_seq1_m.13487_g.13487 | Uncharacterized protein | 0.59 | 1.426E-02 | Down |
| comp5046_c0_seq2_m.18586_g.18586 | TSA: Wollemia nobilis Ref_Wollemi_Transcript_10137_1757 transcribed RNA sequence | 0.576 | 4.714E-05 | Down |
| comp9663_c0_seq2_m.9190_g.9190 | TSA: Wollemia nobilis Ref_Wollemi_Transcript_18719_3499 transcribed RNA sequence | 0.573 | 3.684E-04 | Down |
| comp1919_c0_seq1_m.10436_g.10436 | Putative pectin methylesterase (Fragment) | 0.571 | 1.008E-04 | Down |
| comp1428_c0_seq1_m.11483_g.11483 | Putative arabinogalactan/proline-rich protein | 0.565 | 1.848E-04 | Down |
| comp658_c0_seq2_m.11809_g.11809 | alpha carbonic anhydrase 7-like isoform X2 | 0.553 | 1.664E-04 | Down |
| comp2597_c0_seq1_m.5183_g.5183 | Lipid binding protein | 0.543 | 2.675E-05 | Down |
| comp7343_c0_seq1_m.2913_g.2913 | Cytochrome c-552/DMSO reductase-like | 0.542 | 1.213E-02 | Down |
| comp1523_c0_seq4_m.10456_g.10456 | Pectinesterase | 0.539 | 5.455E-04 | Down |
| comp10477_c0_seq1_m.21_g.21 | mediator of RNA polymerase II transcription subunit 8 isoform X1 | 0.527 | 6.080E-03 | Down |
| comp967_c0_seq1_m.11256_g.11256 | TSA: Wollemia nobilis Ref_Wollemi_Transcript_21687_1028 transcribed RNA sequence | 0.519 | 6.535E-04 | Down |
| comp1116_c0_seq1_m.10894_g.10894 | Lipase 1 | 0.489 | 6.912E-06 | Down |
| comp9596_c0_seq3_m.8033_g.8033 | Peroxidase | 0.486 | 1.138E-04 | Down |
| comp30252_c0_seq1_m.9925_g.9925 | Abscisic acid stress ripening protein homolog | 0.463 | 5.340E-05 | Down |
| comp15825_c0_seq1_m.21106_g.21106 | Glycosyltransferase | 0.458 | 3.798E-05 | Down |
| comp7668_c0_seq1_m.3572_g.3572 | Laccase | 0.458 | 3.409E-05 | Down |
| comp648_c0_seq3_m.10893_g.10893 | GASA2 | 0.443 | 3.488E-06 | Down |
| comp2451_c0_seq2_m.6937_g.6937 | Pectin acetylesterase | 0.373 | 4.039E-05 | Down |
| comp6547_c0_seq1_m.3907_g.3907 | Glycoside hydrolase | 0.363 | 4.540E-06 | Down |
| comp33423_c0_seq1_m.14291_g.14291 | Ferredoxin | 0.278 | 1.954E-06 | Down |
| comp24182_c0_seq1_m.17196_g.17196 | Thaumatin-like protein L2 | 0.27 | 1.582E-06 | Down |
| comp1446_c0_seq6_m.10462_g.10462 | Lipase 1 | 0.246 | 1.295E-06 | Down |
| comp30325_c0_seq1_m.10041_g.10041 | TSA: Wollemia nobilis Ref_Wollemi_Transcript_13084_1284 transcribed RNA sequence | 0.178 | 3.441E-06 | Down |
